# Proliferation, bcl-2 expression and angiogenesis in pituitary adenomas: relationship to tumour behaviour

**DOI:** 10.1054/bjoc.1999.1074

**Published:** 2000-03-21

**Authors:** H E Turner, Zs Nagy, K C Gatter, M M Esiri, J A H Wass, A L Harris

**Affiliations:** Departments of Endocrinology and Neuropathology, Radcliffe Infirmary, Oxford, UK; Department of Pharmacology, University of Oxford, Oxford, UK; Department of Cellular Science and Molecular Angiogenesis Group, ICRF, Institute of Molecular Medicine, John Radcliffe Hospital, Oxford, OX3 9DS, UK

**Keywords:** angiogenesis, proliferation, apoptosis, pituitary tumour, bcl-2

## Abstract

The prediction of pituitary tumour behaviour, in terms of response to treatment from which can be derived optimal management strategies, is a challenge that has been approached using several different means. Angiogenesis in other tumour types has been shown to be correlated with poor response to treatment and tumour recurrence. The aim of this paper is to assess the role of measurements of cell proliferation and angiogenesis in predicting pituitary tumour behaviour. The proliferative capacity of the tumour was assessed using the Ki-67 labelling index (LI) while bcl-2 expression was used to assess anti-apoptotic pathways. The microvessel density (MVD) was assessed using antibodies to CD31 and factor VIII-related antigen, and with biotinylated ulex europaeus agglutinin I. There was no difference between Ki-67 LI and MVD of functionless tumours that recurred and those that did not, but bcl-2 expression was significantly lower in tumours that subsequently regrew. Macroprolactinomas had significantly higher LI than microprolactinomas and than all other tumours. Cell proliferation and angiogenesis were not related, showing that both processes are under different control mechanisms in pituitary tumours. In contrast there was a positive relationship between markers of angiogenesis and bcl-2 expression in prolactinomas, GH-secreting tumours and non-recurrent functionless tumours with higher levels of bcl-2 expression being found in the more vascular tumours. These findings may suggest that angiogenesis is related to the ability of tumour cells to survive rather than their proliferative activity. © 2000 Cancer Research Campaign


British Journal of Cancer (2000) 82(8), 1441–1445
© 2000 Cancer Research Campaign
DOI: 10.1054/ bjoc.1999.1074, available online at http://www.idealibrary.com on IDEAL®

### Proliferation, bcl-2 expression and angiogenesis in pituitary adenomas: relationship to tumour behaviour

**HE Turner[1], Zs Nagy[2,3], KC Gatter[4], MM Esiri[2], JAH Wass[1] and AL Harris[5]**

Departments of [1]Endocrinology and [2]Neuropathology, Radcliffe Infirmary, Oxford, UK; [3]Department of Pharmacology, University of Oxford, Oxford, UK; [4]Department of Cellular Science and [5]Molecular Angiogenesis Group, ICRF, Institute of Molecular Medicine, John Radcliffe Hospital, Oxford, OX3 9DS, UK

**Summary** The prediction of pituitary tumour behaviour, in terms of response to treatment from which can be derived optimal management strategies, is a challenge that has been approached using several different means. Angiogenesis in other tumour types has been shown to be correlated with poor response to treatment and tumour recurrence. The aim of this paper is to assess the role of measurements of cell proliferation and angiogenesis in predicting pituitary tumour behaviour. The proliferative capacity of the tumour was assessed using the Ki-67 labelling index (LI) while bcl-2 expression was used to assess anti-apoptotic pathways. The microvessel density (MVD) was assessed using antibodies to CD31 and factor VIII-related antigen, and with biotinylated ulex europaeus agglutinin I. There was no difference between Ki-67 LI and MVD of functionless tumours that recurred and those that did not, but bcl-2 expression was significantly lower in tumours that subsequently regrew. Macroprolactinomas had significantly higher LI than microprolactinomas and than all other tumours. Cell proliferation and angiogenesis were not related, showing that both processes are under different control mechanisms in pituitary tumours. In contrast there was a positive relationship between markers of angiogenesis and bcl-2 expression in prolactinomas, GH-secreting tumours and non-recurrent functionless tumours with higher levels of bcl-2 expression being found in the more vascular tumours. These findings may suggest that angiogenesis is related to the ability of tumour cells to survive rather than their proliferative activity. © 2000 Cancer Research Campaign

**Keywords**: angiogenesis; proliferation; apoptosis; pituitary tumour; bcl-2

The prediction of pituitary tumour behaviour, in terms of response to treatment from which can be derived optimal management strategies, is a challenge that has been approached using several different means. This paper assesses the role of measurements of cell proliferation and angiogenesis in predicting tumour behaviour. Although metastasis is extremely rare, local recurrence and invasion are important problems and we have investigated whether factors of importance in epithelial cancers are also relevant in these tumours.

There are currently no reliable histological or molecular markers that predict pituitary tumour behaviour and response to treatment. Assessment of cell proliferation by measuring the proportion of cells that have entered the cell cycle has been suggested as a potential means of predicting tumour behaviour (Thapar et al, 1996; Turner and Wass 1999). The MIB-1 labelling index (LI) (proportion of cells in the cell cycle) of hormonally active tumours has been shown to be higher than in endocrinologically inactive adenomas (Thapar et al, 1996) although the reason for this difference is unclear.

Bcl-2, the protein product of the bcl-2 proto-oncogene, inhibits programmed cell death (apoptosis) and gives cells that over-express this protein a survival advantage (Hockenbery, 1992). There has been one previous small study in the pituitary showing that 30% of tumours expressed bcl-2 and suggesting that this may play a role in pituitary tumour pathogenesis through regulation of apoptosis (Wang et al, 1996). Bcl-2 has also been shown to have an inhibitory effect on cell growth (Knowlton et al, 1998).

In many tumour types, angiogenesis (measured as tumour microvessel density (MVD)) has been shown to be correlated with different aspects of tumour behaviour including development of metastasis (Weidner et al, 1991), poor prognosis (Weidner et al, 1992; Weidner, 1996), lower survival (Bochner et al, 1995; Maeda et al, 1995) and recurrence (Gasparini et al, 1994; Frank et al, 1995). MVD has been measured by counting vessels identified by positive immunostaining with antibodies to different vascular endothelial markers. Different antibodies demonstrate different sensitivities for the detection of endothelium. Factor eight-related antigen (F8) stains large vessels but does not label smaller microvessels (Mukai et al, 1980), CD31 stains microvessels but antigen loss can make this unreliable in paraffin-embedded tissue and ulex europaeus agglutinin I (UEAI) stains all microvessels, but also stains the Golgi in some neoplastic cells (Holthofer et al, 1982; Witt and Klessen, 1987; Vermeulen et al, 1996).

Our previous work has demonstrated reduced angiogenesis in pituitary adenomas compared with the normal anterior pituitary (Turner et al, submitted). The aim of this study was to assess the value of the three phenomena: angiogenesis, cell proliferative capacity and anti-apoptotic protein expression in predicting tumour behaviour, as well as to investigate the relationship between tumour angiogenesis, cell proliferation and cell survival capacity.

*Received 1 March 1999*
*Revised 1 September 1999*
*Accepted 11 November 1999*

*Correspondence to*: AL Harris

**Table 1** Antibodies used

| Primary antibody[a] | Pretreatment | Secondary antibody[b] | Colour development |
|---|---|---|---|
| CD31 1/20 (DAKO) | 0.1% trypsin, followed by microwave[c] | Antimouse 1/200 (Insight Biotech) | Horseradish peroxidase streptavidin 1/400 (Dako) and DAB[d] |
| F8 EPOS (DAKO) | 0.1% trypsin | None | DAB[d] |
| UEAI 1/100 (Vector) | None | APAAP complex (Vector) | Fast red (20 minutes) |
| Ki-67 EPOS (DAKO) | Microwave[c] | None | DAB[d] |
| bcl-2 1/20 | Microwave[c] | Antimouse horseradish peroxidase labelled polymer (Envision, Dako) | DAB[d] |

[a]Primary antibodies all applied for 60 min. [b]Secondary antibody applied for 30 min. [c]Microwave in sodium citrate pH 6. [d]DAB, metal-enhanced diaminobenzidine (Pierce and Warriner).

**Table 2** Vascular density and labelling index (Ki-67) of recurrent and non-recurrent functionless tumours

| | CD31M | CD31G | F8M | F8G | UEAIM | UEAIG | Ki67 LI | bcl2 grade |
|---|---|---|---|---|---|---|---|---|
| Non-recurrent tumours | 5.4 (0.2) [34] | 2.7 (0.1) [34] | 5.1 (0.2) [43] | 2.3 (0.1) [43] | 7.4 (0.4) [42] | 2.9 (0.1) [42] | 4.3 (0.53) [40] | 1.1 (0.1) [40] |
| Recurrent first presentation | 5.5 (1.0) [0.3] | 2.4 (0.2) [11] | 5.4 (0.5) [8] | 2.2 (0.3) [8] | 7.7 (0.8) [9] | 3.1 (0.2) [9] | 4.1 (1.0) [11] | 0.2 (0.2) [11] |
| Second presentation | 5.4 (0.3) [14] | 2.4 (0.2) [14] | 5.9 (0.4) [13] | 2.6 (0.2) [14] | 7.4 (0.7) [12] | 2.9 (0.2) [12] | 3.3 (1.0) [12] | 0.4 (0.2) [14] |

Mean (SE) [*n*]. M represents mean Chalkley count, and G represents overall semiquantitative grade.

### MATERIALS AND METHODS

### Specimen collection

A total of 160 surgically removed pituitary adenomas were investigated. There were 46 GH-secreting tumours (28 macroadenomas and 18 microadenomas), seven microprolactinomas, 22 macroprolactinomas, 18 ACTH-secreting tumours (Cushing's disease) and 53 non-functioning pituitary adenomas (28 gonadotrophin positive and 14 negative), 11 of which subsequently recurred. In addition, 14 specimens of recurrent functionless tumours removed following second or third operations for regrowth were studied. There were paired samples of tumour from first and second operations in ten cases. Two of the recurrent tumours had been exposed to pituitary irradiation between the first and second operations. All the tissue was fixed in 4% buffered formalin, dehydrated and embedded in paraffin. Histological examination and immunohistochemistry for anterior pituitary hormones had been performed previously and together with the clinical, biological and radiological data were used to fully characterize each tumour type.

### Immunohistochemistry

The streptavidin–biotin peroxidase complex technique was used for CD31, F8, Ki-67 and bcl-2 and the alkaline phosphatase/anti-alkaline phosphatase (APAAP) method was used for UEAI.

Four-micrometre sections were mounted on aptes (3-aminopropyl triethoxy silane; Sigma)-coated slides, dewaxed and rehydrated. Endogenous peroxidase activity was blocked using 3% hydrogen peroxide where applicable. Non-specific primary antibody binding was blocked using fetal calf serum at a dilution of 1:20 for CD31, UEAI and bcl-2. The details of pretreatment, primary and secondary antibodies and colour reactions are shown in Table 1. The slides were lightly counterstained with haematoxylin.

### Assessment of vascular density and quantification of Ki-67 and bcl-2 labelling

All quantification was performed by a single observer without knowledge of tumour type or size.

Quantification of the Ki-67-labelled cells was performed using an image analysis system (VIDAS 21) (Nagy et al, 1997). Tumour cell nuclear staining was recorded as the percentage of positive cells (LI).

Slides stained with bcl-2 were quantified after examination of the whole tumour section at ×10, using a semiquantitative method on a scale from 0 to 3 (none, low density staining, moderate density staining, dense staining respectively) at ×20 and ×40.

The Chalkley point technique was used to measure microvascular density (Fox et al, 1995). The most vascular area of the tumour was identified at low power as the hot spot. A 25-point Chalkley graticule was orientated so that the maximum number of randomly positioned points seen through the eyepiece at higher power were on or within areas of highlighted vessels. The mean of the counts for the three most vascular areas was recorded. An overall subjective semiquantitative grading system was also used (1 and 2 – low and low moderate, and 3 and 4 – moderate and high vascular density). The counts and grades were made by a single observer, and 20% checked by a second blinded observer. We have previously demonstrated good interobserver reliability (100% concordance for grading into low and high vascular density) and intraobserver reliability $\kappa$ value of 0.6 representing substantial agreement (Landis and Koch, 1977) for vascular counts.

### Statistical analysis

The Statgraphics software package was used. The analysis of variance (ANOVA) test was used for categorical data analysis and regression analysis for comparison of continuous variables. Differences between groups were regarded as significant if $P < 0.05$.

© 2000 Cancer Research Campaign

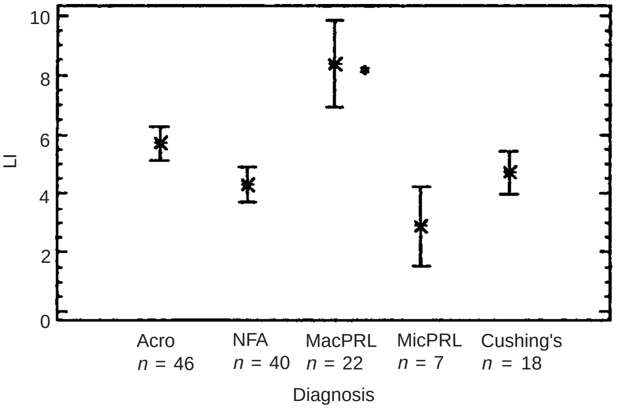

**Figure 1** Comparison of Ki-67 labelling indices in different tumour types. LI: Ki-67 labelling index. Mean values and standard errors. Acro: GH-secreting tumours; NFA: non-functioning tumours; MacPRL: macroprolactinomas; MicPRL: microprolactinomas; Cushing's: ACTH-secreting tumours. *n* is the number of cases in each category. The asterisk indicates the tumour type significantly different ($P < 0.05$) from all other tumour types

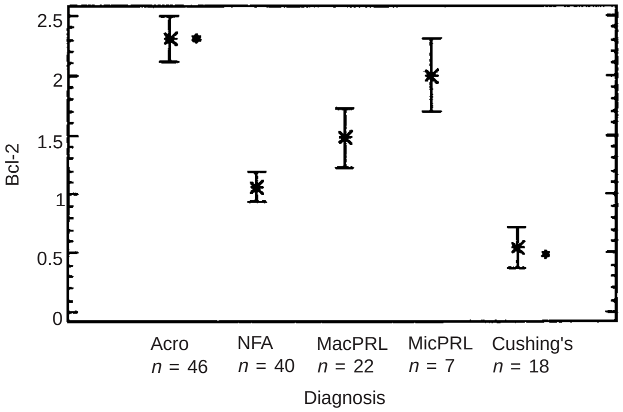

**Figure 2** Comparison of Bcl-2 expression in different tumour types. Mean values and standard errors. Bcl-2: semiquantitative grading of the extent of Bcl-2 labelling. Acro: GH-secreting tumours; NFA: non-functioning tumours; MacPRL: macroprolactinomas; MicPRL: microprolactinomas; Cushing's: ACTH-secreting tumours. *n* is the number of cases in each category. The asterisk indicates the tumour type significantly different ($P < 0.05$) from all other tumour types

### RESULTS

### Ki-67 LI

There was no difference in MVD of functionless tumours that recurred when compared with those that did not (Table 2). Some of the sections of normal pituitary showed one nucleus staining positively for Ki-67, but the majority showed no staining. In contrast, the LI in pituitary adenomas varied between 2.9 and 8.4 depending on tumour type (Figure 1). The LI with Ki-67 was unrelated to whether a non-functioning pituitary tumour recurred or not, and there was no significant difference between the LI of the tumour when the first presentation of a recurrence was compared with the second (Table 2). The LI of macroprolactinomas was significantly higher than the LI of all other tumours $P < 0.05$ (Figure 1). Microprolactinomas had the lowest Ki-67 LI.

**Table 3** Tumour type and bcl-2 expression

| Tumour type | Number (%) – bcl2 positive |
|---|---|
| NFA (non-recurrent) | 31/40 (77.5) |
| NFA (recurrent) | |
| First presentation | 2/11 (18.2) |
| Second presentation | 6/14 (42.9) |
| Macroprolactinoma | 17/22 (77.3) |
| Microprolactinoma | 5/7 (71.4) |
| GH-secreting tumour | 36/46 (78.3) |
| Micro | 12/18 (66.7) |
| Macro | 17/28 (60.7) |
| ACTH-secreting tumour | 9/18 (50) |

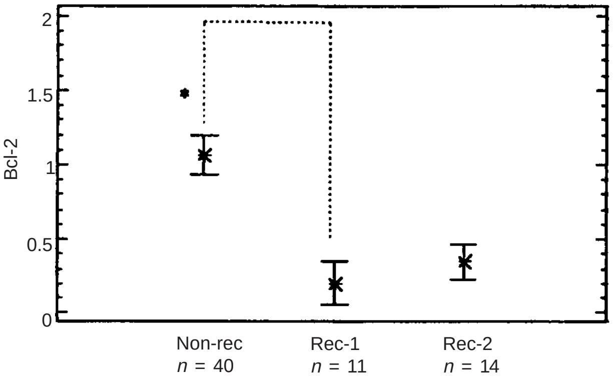

**Figure 3** Bcl-2 expression in recurrent and non-recurrent functionless tumours. Bcl-2: semiquantitative grading of the extent of Bcl-2 labelling. Non-rec: non-recurrent functionless pituitary adenomas; Rec-1: functionless pituitary adenomas that subsequently recurred – first presentation; Rec-2: recurrent functionless pituitary adenomas – presentation of recurrence. Dotted line and asterisk indicate statistically significant difference ($P < 0.05$)

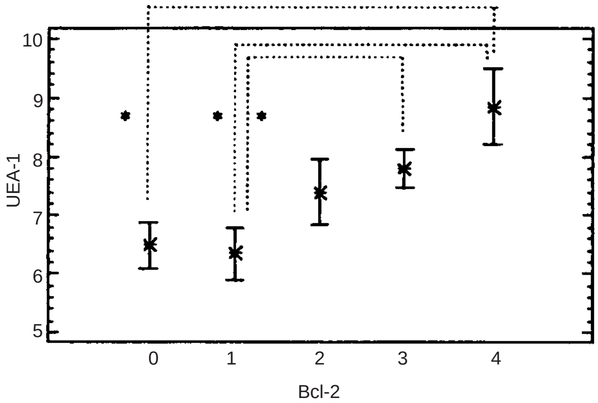

**Figure 4** Relationship between the vascular density and Bcl-2 expression in pituitary tumours. Note *y*-axis is truncated. UEA1: vascular density as measured by UEA1 staining; Bcl-2: semiquantitative grading of the extent of Bcl-2 labelling. Dotted lines and asterisks indicate statistically significant differences ($P < 0.05$)

### Relationship between Ki-67 LI and bcl-2 expression or vascular density

There was no association between Ki-67 LI and vascular density or bcl-2 expression for any tumour type studied. The relationship of MVD to tumour type, behaviour and size have been previously reported (Turner et al, submitted).

© 2000 Cancer Research Campaign

### bcl-2 expression

Normal pituitary did not show positive immunostaining for bcl-2. However, bcl-2 was expressed by over half of the tumours studied with the exception of functionless tumours that recurred (Table 3). bcl-2 grade was significantly higher in GH-secreting tumours compared to ACTH-secreting tumours, NFA and macroprolactinomas. Excluding recurrent functionless tumours, ACTH-secreting tumours were of a significantly lower bcl-2 grade than all other tumours (Figure 2). There was no significant difference in bcl-2 grade between microprolactinomas and macroprolactinomas.

The bcl-2 grade was significantly higher in functionless tumours that did not recur than those that did (Figure 3).

### bcl-2 expression and angiogenesis bcl-2 expression was significantly associated with MVD, with higher bcl-2 expression found in the more vascular tumours (Figure 4). Examination of adjacent sections showed that bcl-2 expression was not necessarily associated with vascular hot spots.

### DISCUSSION

The lack of association between MVD and functionless tumour recurrence indicates that angiogenesis is unlikely to be a major factor in determining which of these tumours regrow. It suggests that other factors such as the amount of residual tumour remaining after surgery or perhaps alterations in cell cycle control (Jin et al, 1997) are more important. It is clear that MVD cannot be used to predict which tumour may subsequently recur and therefore require prophylactic radiotherapy. In contrast to some studies of cell proliferation that have suggested that the Ki-67 LI concords with recurrence after treatment (Knosp et al, 1989; Abe et al, 1997), our data show that the Ki-67 LI as a reflection of tumour proliferation is not useful to predict subsequent behaviour of functionless tumours. The definition of recurrent and non-recurrent tumours may be a confounding factor as pre-emptive radiotherapy may be given to avoid tumour regrowth and recurrence. Inadequate duration of follow-up may inadvertently place a recurrent tumour in the non-recurrent group and finally much of what is defined as recurrence may simply be regrowth of a slowly growing tumour in a location with difficult surgical access.

The Ki-67 LI of macroprolactinomas was significantly higher than all other tumours, in keeping with the fact that they often behave more aggressively than other benign pituitary tumours and often show local invasion. The significant difference between Ki-67 LI of macroprolactinomas and microprolactinomas is in support of the data from Delgrange et al (1997) who demonstrated that microadenomas producing prolactin had a significantly lower Ki-67 LI than macroadenomas. Our data showed no relationship between tumour size and LI in any other tumour type, confirming the findings of other studies (Knosp et al, 1989; Thapar et al, 1996; Turner and Wass, 1999), and suggesting that other factors such as cell cycle arrest, duration of the cell cycle or the rate of cell death are important in determining tumour size.

The lack of an association between Ki-67 LI and MVD in pituitary tumours is not entirely surprising as the LI is simply a measure of the proportion of cells that have entered the cell cycle and gives no idea of the speed of progress through the cycle and therefore tumour growth, which in pituitary adenomas is usually slow. Our data are in support of work in breast cancer and colorectal cancer where intratumoural MVD measured using anti-CD34 and the LI measured using Ki-67 were not related suggesting that angiogenesis and tumour cell proliferation may be regulated by different means (Fox et al, 1993; Vartanian and Weidner, 1994; Vermeulen et al, 1997). In theory, it might be expected that MVD could limit tumour growth by limiting cell proliferation although this is clearly not the case in the pituitary. It has been suggested that the lack of correlation may be because the MVD is more indicative of the apoptotic rather than the proliferative fraction of cells (Vermeulen et al, 1997).

In the only previous study looking at bcl-2 expression in pituitary tumours, bcl-2 was demonstrated using immunohistochemistry to be present in 30% of tumours but not in functionless tumours or normal tissue. Our data have shown that bcl-2 expression is found in over 50% tumours and in particular that 2/3 of the functionless tumours express bcl-2. The differences between the two studies may be related to differences in tissue fixation, numbers of tumours studied, antibodies or immunohistochemistry technique (Envision). This finding supports the suggestion that bcl-2 expression may be an early event in pituitary tumorigenesis leading to indolent tumour growth and cell survival despite slow rates of cell proliferation as bcl-2 expression is known to inhibit cell proliferation (Knowlten et al, 1998). It is also consistent with the fact that apoptosis is an apparently rare event in pituitary tumours. The positive association between bcl-2 expression and MVD suggests that the control of angiogenesis and programmed cell death may be related – perhaps a switch to an angiogenic phenotype and bcl-2 expression are both early events in pituitary tumorigenesis or that non-cycling but surviving cells support angiogenesis. The important pro-angiogenic growth factor, vascular endothelial growth factor (VEGF), has been shown to be present in endocrine tissue (Christofori et al, 1995; Fan and Iseki, 1998; Terris et al, 1998) as well as in tumours. It has been suggested that VEGF may be of particular importance in endocrine tissue, as it also maintains endothelial permeability allowing direct access to the bloodstream. It has recently been demonstrated that VEGF not only stimulates endothelial proliferation, but also prolongs endothelial cell survival by inducing expression of bcl-2, suggesting that part of the angiogenic activity of VEGF may be related to protection of endothelial cells from apoptosis (Nor et al, 1999).

It is therefore perhaps surprising that bcl-2 expression was lower in recurrent tumours than non-recurrent. Although bcl-2 expression may protect from apoptosis, it also inhibits cell growth, and in breast and lung cancer bcl-2 expression is associated with less aggressive tumour behaviour (Fontanini et al, 1995; Berado et al, 1998; Charpin et al, 1998). It is therefore likely that a reduction in bcl-2 expression reflects a change in another pathway that promotes survival and aggressive behaviour, allowing bcl-2 to be down-regulated. The relationship between bcl-2 and angiogenesis has recently been explored by Carmaliet and colleagues who showed that bcl-2 is regulated by hypoxia. Increased hypoxia inducible factor-1 (HIF-1$\alpha$) expression in response to reduced oxygen tension leads to suppression of bcl-2 along with increased VEGF and angiogenesis (Carmaliet et al, 1998). The lower expression of bcl-2 in recurrent tumours may thus be a reflection of hypoxia-induced increased HIF-1$\alpha$ expression and secondary suppression of bcl-2 expression. The positive association between MVD and bcl-2 expression in the other pituitary tumour types is difficult to reconcile with the above findings but it has been suggested that the role of HIF-1$\alpha$ in apoptosis may depend on cell

© 2000 Cancer Research Campaign type, genotype or cell density (Carmaliet et al, 1998). Wild-type p53 is a known inhibitor of tumour angiogenesis (Dameron et al, 1994). Further steps in pituitary tumour progression may involve inactive or mutant p53 expression which inhibits HIF-1-stimulated transcription (Blagosklonny et al, 1998). It is known that only very aggressive pituitary tumours express p53 (Thapar et al, 1996*b*). A reciprocal relationship between p53 expression and bcl-2 has been demonstrated in other tumour types, where only very aggressive tumours express p53 (Fontanini et al, 1995).

In conclusion, we have shown that MVD and bcl-2 expression in pituitary tumours are associated, suggesting that angiogenesis may be linked to the ability of cells to survive. There was no relationship between MVD and Ki-67 LI, suggesting that angiogenesis and proliferation are regulated by different means. We have demonstrated that neither angiogenesis nor cell proliferation were predictive of subsequent tumour recurrence but shown that bcl-2 expression was significantly lower in tumours that regrew than those that did not. Cell proliferation as measured using the Ki-67 LI was significantly higher in macroprolactinomas than other tumours, in keeping with their known clinical behaviour. It will be useful to study more recurrent and non-recurrent tumours as well as some malignant tumours to further investigate the relationship between bcl-2 expression, p53, hypoxia and MVD.

### REFERENCES

Abe T, Sanno N, Osamura YR and Matsumoto K (1997) Proliferative potential in pituitary adenomas: measurement by monoclonal antibody MIB-1. *Acta Neurochir* **139**: 613–618

Berardo MD, Elledge RM, de Moor C, Clark GM, Osborne CK and Allred DC (1998) bcl-2 and apoptosis in lymph node positive breast carcinoma. *Cancer* **82**: 1296–1302

Blagosklonny MV, An WG, Romanova LY, Trepel J, Fojo T and Neckers L (1998) p53 inhibits hypoxia-inducible factor-stimulated transcription. *J Biol Chem* **273**: 11995–11998

Bochner BH, Cote RJ, Weidner N, Groshen S, Chen SC, Skinner DG et al (1995) Angiogenesis in bladder cancer: relationship between microvessel density and tumour prognosis. *J Natl Cancer Inst* **87**: 1603–1612

Carmeliet P, Dor Y, Herbert JM, Fukumura D, Brusselmans K, Dewerchin M, Neeman M, Bono F, Abramovitch R, Maxwell P, Koch CJ, Ratcliffe P, Moons L, Jain RK, Collen D and Keshet E (1998) Role of HIF-1 alpha in hypoxia-mediated apoptosis, cell proliferation and tumour angiogenesis. *Nature* **394**: 485–489

Charpin C, Garcia S, Bonnier P, Martini F, Andrac L, Horschowski N, Lavaut MN and Allasia C (1998) bcl-2 automated and quantitative immunocytochemical assays in breast carcinomas: correlation with 10-year follow-up. *J Clin Oncol* **16**: 2025–2031

Christofori G, Naik P and Hanahan D (1995) Vascular endothelial growth factor and its receptors, flt-1 and flk-1, are expressed in normal pancreatic islets and throughout islet cell tumorigenesis. *Mol Endocrinol* **9**: 1760–1770

Dameron KM, Volpert OV, Tainsky MA and Bouck N (1994) Control of angiogenesis in fibroblasts by p53 regulation of thrombospondin-1. *Science* **265**: 1582–1584

Delgrange E, Trouillas J, Maiter D, Donckier J and Turniaire J (1997) Sex-related difference in the growth of prolactinomas: a clinical and proliferation marker study. *J Clin Endocrinol Metab* **82**: 2102–2107

Fan L and Iseki S (1998) Immunohistochemical localization of vascular endothelial growth factor in the endocrine glands of the rat. *Arch Histol Cytol* **61**: 17–28

Fontanini G, Vignati S, Bigini D, Mussi A, Lucchi M, Angeletti CA, Basolo F and Bevilacqua G (1995) Bcl-2 protein: a prognostic factor inversely correlated to p53 in non-small-cell lung cancer. *Br J Cancer* **71**: 1003–1007

Fox SB, Gatter KC, Bicknell R, Going JJ, Stanton P, Cooke TG and Harris AL (1993) Relationship of endothelial cell proliferation to tumor vascularity in human breast cancer. *Cancer Res* **53**: 4161–4163

Fox SB, Leek RD, Weekes MP, Whitehouse RM, Gatter KC and Harris AL (1995) Quantification and prognostic value of breast cancer angiogenesis: Chalkley count and computer image analysis. *J Pathol* **177**: 275–283

Frank RE, Saclarides TJ, Leurgans S, Speziale NJ, Drab EA and Rubin DB (1995) Tumor angiogenesis as a predictor of recurrence and survival in patients with node-negative colon cancer. *Ann Surg* **222**: 695–690

Gasparini G, Weidner N, Bevilacqua P, Maluta S, Palma PD, Caffo O, Barbareschi M, Boracchi P, Marubini E and Pozza F (1994) Tumour microvessel density, p53 expression, tumour size and peritumoural lymphatic vessel invasion are relevant prognostic markers in node-negative breast carcinoma. *J Clin Oncol* **12**: 454–466

Holthofer H, Virtanen I, Kariniemi AL, Hormia M, Linder E and Miettinen A (1982) Ulex europaeus I lectin as a marker for vascular endothelium in human tissues. *Lab Invest* **47**: 60–65

Hockenbery DM (1992) The bcl-2 oncogene and apoptosis. *Semin Immunol* **4**: 413–420

Jin L, Qian X, Kulig E, Sanno N, Scheithauer BW, Kovacs K, Young WF Jr and Lloyd RV (1997) Transforming growth factor-beta, transforming growth factor-beta receptor II, and p27 Kip1 expression in nontumorous and neoplastic human pituitaries. *Am J Pathol* **151**: 509–510

Knosp E, Kitz K and Perneczky A (1989) Proliferation activity in pituitary adenomas: measurement by monoclonal antibody Ki-67. *Neurosurgery* **25**: 927–930

Knowlton K, Mancini M, Creason S, Morales C, Hockenbery D and Anderson BO (1998) Bcl-2 slows in vitro breast cancer growth despite its antiapoptotic effect. *J Surg Res* **76**: 22–26

Landis JR and Koch GC (1977) The measurement of observer agreement for categorical data. *Biometrics* **33**: 159–174

Maeda K, Chung Y-S, Takasuka S, Ogawa Y, Sawada T, Yamashita Y et al (1995) Tumour angiogenesis as a predictor of recurrence in gastric carcinoma. *J Clin Oncol* **13**: 477–481

Mukai K, Rosai J and Burgdorf WHC (1980) Localisation of factor VIII-related antigen in vascular endothelial cells using an immunoperoxidase method. *Am J Surg Pathol* **4**: 273

Nagy ZS, Esiri MM and Smith AD (1997) Expression of cell division markers in the hippocampus in Alzheimer's disease and other neurodegenerative conditions. *Acta Neuropathol* **93**: 294–300

Nor JE, Christensen J, Mooney DJ, Polverini PJ (1999) Vascular endothelial growth factor (VEGF)-mediated angiogenesis is associated with enhanced endothelial cell survival and induction of Bcl-2 expression. *Am J Pathol* **154**: 375–384

Terris B, Scoazec JY, Rubbia L, Bregeaud L, Pepper MS, Ruszniewski P, Belghiti J, Flejou J and Degott C (1998) Expression of vascular endothelial growth factor in digestive neuroendocrine tumours. *Histopathology* **32**: 133–138

Thapar K, Kovacs K, Scheithauer BW, Stefaneanu L, Horvath E, Pernicone PJ, Murray D and Laws ER (1996*a*) Proliferative activity and invasiveness among pituitary adenomas and carcinomas: an analysis using the MIB-1 antibody. *Neurosurgery* **38**: 99–107

Thapar K, Scheithauer BW, Kovacs K, Pernicone PJ and Laws ER Jr (1996*b*) p53 expression in pituitary adenomas and carcinomas: correlation with invasiveness and tumor growth fractions. *Neurosurgery* **38**: 763–770; discussion 770–771

Turner HE and Wass JAH (1999) Are markers of proliferation valuable in the histological assessment of pituitary tumours? *Pituitary* **1**: 147–151

Vartanian RK and Weidner N (1994) Correlation of intratumoural endothelial cell proliferation with microvessel density (tumour angiogenesis) and tumour cell proliferation in breast carcinoma. *Am J Pathol* **144**: 1188–1194

Vermeulen PB, Gasparini G, Fox SB, Toi M, Martin L, McCulloch P, Pezella F, Viale G, Weidner N, Harris AL and Dirix LY (1996) Quantification of angiogenesis in solid human tumours: an international consensus on the methodology and criteria of evaluation. *Eur J Cancer* **32A**: 2474–2484

Vermeulen PB, Dirix LY, Libura J, Vanhoolst IF, Van Marck E and Oosterom AT (1997) Correlation of the fractions of proliferating tumour and endothelial cells in breast and colorectal adenocarcinoma is independent of tumour histiotype and microvessel density. *Microvasc Res* **54**: 88–92

Wang DG, Johnston CF, Atkinson AB, Heaney AP, Mirakhur M and Buchanan KD (1996) Expression of bcl-2 oncoprotein in pituitary tumours: comparison with c-myc. *JClinPathol* **49**: 795–797

Weidner N, Semple JP, Welch WR and Folkman J (1991) Tumour angiogenesis and metastasis: correlation in invasive breast carcinoma. *N Engl J Med* **324**: 1–8

Weidner N, Folkman J, Pozza F, Bevilacqua P, Allred EN, Moore DH, Meli S and Gasparini G (1992) Tumour angiogenesis: a new significant and independent prognostic indicator in early stage breast carcinoma. *J Natl Cancer Inst* **84**: 1875–1887

Weidner N (1996) Intratumoural vascularity as a prognostic factor in cancers of the urogenital tract. *Eur J Cancer* **32A**: 2506–2512

Witt M and Klessen Ch (1987) Galactose and fucose binding sites in anterior pituitary cells of the rat: detection by means of biotinylated lectins. *Folia Histochem Cytobiol* **25**: 115–118

© 2000 Cancer Research Campaign 

## References

[PDF_00441] Abe T., Sanno N., Osamura Y. R., Matsumoto K. (1997). Proliferative potential in pituitary adenomas: measurement by monoclonal antibody MIB-1.. Acta Neurochir (Wien).

[PDF_00444] Berardo M. D., Elledge R. M., de Moor C., Clark G. M., Osborne C. K., Allred D. C. (1998). bcl-2 and apoptosis in lymph node positive breast carcinoma.. Cancer.

[PDF_00447] Blagosklonny M. V., An W. G., Romanova L. Y., Trepel J., Fojo T., Neckers L. (1998). p53 inhibits hypoxia-inducible factor-stimulated transcription.. J Biol Chem.

[PDF_00450] Bochner B. H., Cote R. J., Weidner N., Groshen S., Chen S. C., Skinner D. G., Nichols P. W. (1995). Angiogenesis in bladder cancer: relationship between microvessel density and tumor prognosis.. J Natl Cancer Inst.

[PDF_00453] Carmeliet P., Dor Y., Herbert J. M., Fukumura D., Brusselmans K., Dewerchin M., Neeman M., Bono F., Abramovitch R., Maxwell P. (1998). Role of HIF-1alpha in hypoxia-mediated apoptosis, cell proliferation and tumour angiogenesis.. Nature.

[PDF_00458] Charpin C., Garcia S., Bonnier P., Martini F., Andrac L., Horschowski N., Lavaut M. N., Allasia C. (1998). bcl-2 automated and quantitative immunocytochemical assays in breast carcinomas: correlation with 10-year follow-up.. J Clin Oncol.

[PDF_00462] Christofori G., Naik P., Hanahan D. (1995). Vascular endothelial growth factor and its receptors, flt-1 and flk-1, are expressed in normal pancreatic islets and throughout islet cell tumorigenesis.. Mol Endocrinol.

[PDF_00465] Dameron K. M., Volpert O. V., Tainsky M. A., Bouck N. (1994). Control of angiogenesis in fibroblasts by p53 regulation of thrombospondin-1.. Science.

[PDF_00468] Delgrange E., Trouillas J., Maiter D., Donckier J., Tourniaire J. (1997). Sex-related difference in the growth of prolactinomas: a clinical and proliferation marker study.. J Clin Endocrinol Metab.

[PDF_00471] Fan L., Iseki S. (1998). Immunohistochemical localization of vascular endothelial growth factor in the endocrine glands of the rat.. Arch Histol Cytol.

[PDF_00474] Fontanini G., Vignati S., Bigini D., Mussi A., Lucchi M., Angeletti C. A., Basolo F., Bevilacqua G. (1995). Bcl-2 protein: a prognostic factor inversely correlated to p53 in non-small-cell lung cancer.. Br J Cancer.

[PDF_00477] Fox S. B., Gatter K. C., Bicknell R., Going J. J., Stanton P., Cooke T. G., Harris A. L. (1993). Relationship of endothelial cell proliferation to tumor vascularity in human breast cancer.. Cancer Res.

[PDF_00480] Fox S. B., Leek R. D., Weekes M. P., Whitehouse R. M., Gatter K. C., Harris A. L. (1995). Quantitation and prognostic value of breast cancer angiogenesis: comparison of microvessel density, Chalkley count, and computer image analysis.. J Pathol.

[PDF_00483] Frank R. E., Saclarides T. J., Leurgans S., Speziale N. J., Drab E. A., Rubin D. B. (1995). Tumor angiogenesis as a predictor of recurrence and survival in patients with node-negative colon cancer.. Ann Surg.

[PDF_00486] Gasparini G., Weidner N., Bevilacqua P., Maluta S., Dalla Palma P., Caffo O., Barbareschi M., Boracchi P., Marubini E., Pozza F. (1994). Tumor microvessel density, p53 expression, tumor size, and peritumoral lymphatic vessel invasion are relevant prognostic markers in node-negative breast carcinoma.. J Clin Oncol.

[PDF_00494] Hockenbery D. M. (1992). The bcl-2 oncogene and apoptosis.. Semin Immunol.

[PDF_00491] Holthöfer H., Virtanen I., Kariniemi A. L., Hormia M., Linder E., Miettinen A. (1982). Ulex europaeus I lectin as a marker for vascular endothelium in human tissues.. Lab Invest.

[PDF_00496] Jin L., Qian X., Kulig E., Sanno N., Scheithauer B. W., Kovacs K., Young W. F., Lloyd R. V. (1997). Transforming growth factor-beta, transforming growth factor-beta receptor II, and p27Kip1 expression in nontumorous and neoplastic human pituitaries.. Am J Pathol.

[PDF_00500] Knosp E., Kitz K., Perneczky A. (1989). Proliferation activity in pituitary adenomas: measurement by monoclonal antibody Ki-67.. Neurosurgery.

[PDF_00503] Knowlton K., Mancini M., Creason S., Morales C., Hockenbery D., Anderson B. O. (1998). Bcl-2 slows in vitro breast cancer growth despite its antiapoptotic effect.. J Surg Res.

[PDF_00506] Landis J. R., Koch G. G. (1977). The measurement of observer agreement for categorical data.. Biometrics.

[PDF_00508] Maeda K., Chung Y. S., Takatsuka S., Ogawa Y., Sawada T., Yamashita Y., Onoda N., Kato Y., Nitta A., Arimoto Y. (1995). Tumor angiogenesis as a predictor of recurrence in gastric carcinoma.. J Clin Oncol.

[PDF_00511] Mukai K., Rosai J., Burgdorf W. H. (1980). Localization of factor VIII-related antigen in vascular endothelial cells using an immunoperoxidase method.. Am J Surg Pathol.

[PDF_00514] Nagy Z., Esiri M. M., Smith A. D. (1997). Expression of cell division markers in the hippocampus in Alzheimer's disease and other neurodegenerative conditions.. Acta Neuropathol.

[PDF_00517] Nör J. E., Christensen J., Mooney D. J., Polverini P. J. (1999). Vascular endothelial growth factor (VEGF)-mediated angiogenesis is associated with enhanced endothelial cell survival and induction of Bcl-2 expression.. Am J Pathol.

[PDF_00520] Terris B., Scoazec J. Y., Rubbia L., Bregeaud L., Pepper M. S., Ruszniewski P., Belghiti J., Fléjou J., Degott C. (1998). Expression of vascular endothelial growth factor in digestive neuroendocrine tumours.. Histopathology.

[PDF_00523] Thapar K., Kovacs K., Scheithauer B. W., Stefaneanu L., Horvath E., Pernicone P. J., Murray D., Laws E. R. (1996). Proliferative activity and invasiveness among pituitary adenomas and carcinomas: an analysis using the MIB-1 antibody.. Neurosurgery.

[PDF_00527] Thapar K., Scheithauer B. W., Kovacs K., Pernicone P. J., Laws E. R. (1996). p53 expression in pituitary adenomas and carcinomas: correlation with invasiveness and tumor growth fractions.. Neurosurgery.

[PDF_00530] Turner H. E., Wass J. A. (1999). Are markers of proliferation valuable in the histological assessment of pituitary tumours?. Pituitary.

[PDF_00532] Vartanian R. K., Weidner N. (1994). Correlation of intratumoral endothelial cell proliferation with microvessel density (tumor angiogenesis) and tumor cell proliferation in breast carcinoma.. Am J Pathol.

[PDF_00539] Vermeulen P. B., Dirix L. Y., Libura J., Vanhoolst I. F., Van Marck E., Van Oosterom A. T. (1997). Correlation of the fractions of proliferating tumor and endothelial cells in breast and colorectal adenocarcinoma is independent of tumor histiotype and microvessel density.. Microvasc Res.

[PDF_00535] Vermeulen P. B., Gasparini G., Fox S. B., Toi M., Martin L., McCulloch P., Pezzella F., Viale G., Weidner N., Harris A. L. (1996). Quantification of angiogenesis in solid human tumours: an international consensus on the methodology and criteria of evaluation.. Eur J Cancer.

[PDF_00543] Wang D. G., Johnston C. F., Atkinson A. B., Heaney A. P., Mirakhur M., Buchanan K. D. (1996). Expression of bcl-2 oncoprotein in pituitary tumours: comparison with c-myc.. J Clin Pathol.

[PDF_00548] Weidner N., Folkman J., Pozza F., Bevilacqua P., Allred E. N., Moore D. H., Meli S., Gasparini G. (1992). Tumor angiogenesis: a new significant and independent prognostic indicator in early-stage breast carcinoma.. J Natl Cancer Inst.

[PDF_00552] Weidner N. (1996). Intratumoral vascularity as a prognostic factor in cancers of the urogenital tract.. Eur J Cancer.

[PDF_00546] Weidner N., Semple J. P., Welch W. R., Folkman J. (1991). Tumor angiogenesis and metastasis--correlation in invasive breast carcinoma.. N Engl J Med.

[PDF_00554] Witt M., Klessen C. (1987). Galactose and fucose binding sites in anterior pituitary cells of the rat. Detection by means of biotinylated lectins.. Folia Histochem Cytobiol.

